# 

**Published:** 1896-06

**Authors:** 


					CONTENTS.
TAGE I
The Clinical Significance of the
Human Hand.
By Arthur S.
Wohlmann, M.D., B.S. Lond.
117
Induced Premature Labour in Cer-
tain Diseases of the Mother not
Obstructing Delivery
(cont.). By
J. G. Swayne, M.D. Lond.
126
Chloroform or Ether ?
By John
Freeman, F.R.C.S. Ed.
137
Dermoid Cyst of a Branchial Cleft.
By Thomas Carwardine, M.B.,
M.S. Lond., F.R.C.S. Eng
149
A Case of Ligature and Division of
the Vasa Deferentia for Pros-
tatic Hypertrophy.
By C. W. J.
Brasher, M.R.C.S. Eng., L.R.C.P.
Lond
ISO
Progress of the Medical Sciences:
Medicine.
By George Parker, M.D.
1SS
Surgery.
By C. A. Morton
161
Obstetrics.
TV/T
By Walter C. Swayne,
M.D.
168
Reviews of Books
173
Notes on Preparations for the Sick
187
Meetings of Societies
191
The Library of the Bristol Medico-
Chirurgical Society 206
Local Medical Notes
209
International Congress of Derma-
tology
210
International Congress of Gynae-
cology and Obstetrics
210
Scraps
211

				

